# Enzyme activities and gene expression of starch metabolism provide insights into grape berry development

**DOI:** 10.1038/hortres.2017.18

**Published:** 2017-05-10

**Authors:** Xudong Zhu, Chaobo Zhang, Weimin Wu, Xiaopeng Li, Chuan Zhang, Jinggui Fang

**Affiliations:** 1 College of Horticulture, Nanjing Agricultural University, No 1 weigang, Nanjing 210095, China; 2 Institute of Horticulture, Jiangsu Academy of Agricultural Sciences, No 50 Zhongling road, Nanjing 210014, China

## Abstract

Grapes are categorized as a non-climacteric type of fruit which its ripening is not associated to important rises in respiration and ethylene synthesis. The starch metabolism shares a certain role in the carbohydrate metabolic pathways during grape berry development, and is regarded as an important transient pool in the pathway of sugar accumulation. However, the comprehensive role of starch and its contribution to the quality and flavor of grape berry have not been explored thoroughly. In this study, the expression levels of genes enzyme activities and carbohydrate concentrations related to starch metabolism, were analyzed to understand the molecular mechanism of starch accumulation during grape berry development. The results indicated that starch granules in grape berry were located at the chloroplast in the sub-epidermal tissues, acting as the temporary reserves of photosynthetic products to meet the needs for berry development, and relatively high starch contents could be detected at véraison stage. Moreover, both ADP-glucose pyrophosphorylase (EC 2.7.7.27) and sucrose phosphate synthase (EC 2.3.1.14) involved in starch synthesis displayed elevated gene expression and enzymes activities in the sub-epidermal tissue, while α- and β-amylases involved in its degradation were highly transcribed and active in the central flesh, explaining the absence of starch in this last tissue. Change in the gene expression and activities of ADP-glucose pyrophosphorylase, β-amylase and sucrose phosphate synthase revealed that they were regulated by the circadian rhythms in the fruitlets compared with those in the leaves. Both the morphological, enzymological and transcriptional data in this study provide advanced understandings on the function of starch during berry development and ripening that are so important for berry quality. This study will further facilitate our understanding of the sugar metabolism in grape berry as well as in other plant species.

## Introduction

Grapes are among the most widely cultivated and important commercial fruit crops around the world. Starch is pivotal for the plant growth and development, and its accumulation may influence the final soluble sugar concentration at ripe stage, in many fruits like apple and banana. Even grape berry contained no significant amounts of starch compared the leaves and roots,^1^ starch may influence the sugar partitioning and utilization of grape berry under a more complex mechanism.^2^ The quality trait of the grape berry about the sugar concentration is becoming more and more important for consumers, and the selection of new grapevine cultivars with enhanced sugar content is still a key major goal of breeding programs.^3,4^ In order to regulate the sugar accumulation in grape berry with management practice and even accelerate the development of elite cultivars, a better understanding of sugar metabolism and storage in grape is required.

Carbohydrates are biosynthesized in the source tissues (leaves) and translocated to the sink tissues (roots, flowers, fruits) in most plant species in the form of soluble sugars to sustain heterotrophic metabolism, including pineapple, cherry, fig, strawberry, citrus fruits and grape. Growth and development of plants is accompanied by changes in the relations of source/sink tissues.^5^ Grape berries accumulate different kinds of soluble sugar, mainly sucrose, glucose and fructose during ripening. Glucose and fructose are the two predominant sugars of the grape berry at maturity. Whereas, the grape starch metabolism is a highly complex, dynamic process, which is represented by the contemporaneous synthesis and degradation of starch.^6–8^ Many fruit crops such as apple, banana and kiwifruit contain large contents of starch before the onset of ripening, but grape was reported to have a low quantity of starch granules present in the fruitlets at anthesis period,^6^ then the starch presented only in the epidermis and a few layers of sub-epidermal cells at maturity period, and finally disappeared in most tissues.^1^ Many studies have already been conducted on the developmental changes of sugar metabolism in grapevine (*Vitis vinifera*).^9–11^ These studies focused on the metabolic changes of glucose, fructose and even sucrose which total account for about 99 percent of the entire carbohydrate content of grape berry, regulated both the sugariness of the berry and the ethanol content of the wine produced through the fermentation of grapes. Starch may represent an important intermediate in the global pathway of sugar accumulation in grape berry. Some reports on the role of starch during the grape berry development have indicated that the starch reserves in plastid are transported to produce new hexose (glucose and fructose).^9^ However, it is still necessary to have more insights into the regulation network about starch metabolism pathways at different periods of grape development, and in the circadian rhythm, which will provide profound understandings in the developmental regulatory mechanism of starch is coordinated with other sugars, and the information will benefit both scientific motivation and economic interest.

The circadian fluctuation of transient accumulation and degradation of starch in source leaves has been studied extensively in many plants.^12–15^ It was accepted that ADP-glucose pyrophosphorylase (AGPase), granule bounded starch syntheses (GBSS), alpha-amylase (AMY) and beta-amylase (BMY) played a crucial role in controlling starch synthesis and degradation in the tubers, germinating cereal seeds and roots.^16–20^ It is often suggested that AGPase, GBSS and BMY played a similar role in the degradation of transitory starch in chloroplasts.^21–23^ John *et al.*^24^ demonstrated that the low activities of starch synthase and adenosine diphosphate glucose pyrophosphorylase may result in the shortage of starch in grape berries. Xie *et al.*^25^ found that during the first rapid growth phase of the grape berry, cells had more abundant mitochondria, endoplasmic reticulum, multivesicular bodies, vesicles, and plastids than in control fruit under root restriction. However, the role that starch played in grape berry still need further study.

In this study, transmission electron microscopy was used to locate where the starch granule in grape berry tissues resides. The carbohydrate contents, the activities of key enzymes and the expression patterns of genes related to starch-sucrose metabolism in grape berry were also examined to investigate the changes of starch metabolism occurring in the developmental process of grape berry. The results may provide a background of relationship between starch metabolism and berry quality of *V. vitifera*, which may be beneficial for our understanding the process of quality formation and accelerating the breeding program in the future.

## Materials and methods

### Plant materials

Field-grown grapevines cv. ‘Yongyou 1 Hao’ (*Vitis vinifera*×*Vitis labrusca*) (red cultivar) have been grown for 8 years from the grapevine germplasm repository (established by Institute of Horticulture, Jiangsu Academy of Agricultural Sciences, Nanjing, China). Vines were grown with 3.0×1.5 m spacing, east-west direction and suitable plant protection measures. To select representative samples in the vineyard, we sampled based on the method of Boulton *et al.*^26^ The vineyard was divided into three plots from which three grapevine trees per plot were selected for tissue collection.

To investigate the circadian rhythm of sugar metabolism, leaves and fruitlets from the same node were sampled at 14 DAA (days after anthesis) from the outer southern canopies at 9, 13, 17, 21, 1 and 5 O’clock in the daytime. The anthesis (flowering) was described as the time when about 50% flowers are fully open. For exploring the difference between the skin and flesh on starch metabolism during fruit development, three berries from top, middle and bottom of the berry bunch were sampled at eight stages (7, 14, 21, 28, 42, 56, 70 and 84 DAA) and immediately divided into skin and flesh employing a fine edged scalpel. These seeds were discarded, and berries mixed to one sample at per point.

While, the fruitlets at 7 and 14 DAA were too small to separate the skin and flesh, so the fruitlets at 7 and 14 DAA can be considered respectively as both the skin and flesh at 7 and 14 DAA, that is, in the subsequent results, the enzyme activities and gene expression between the skin and flesh at 7 and 14 DAA were the same. For carbohydrate analysis as well as enzyme and RNA extractions, each tissue from individual sampling points and dates was immediately frozen using the liquid nitrogen and then stored in −70 °C freezer until needed. Each data presented here was the mean value of three replicate samples and each sample was measured three times.

### Transmission electron microscopy

To investigate the changes of starch granules in the skin and flesh cells during grape berry development, the skin and flesh tissues were prepared for electron microscopy. Tissue preparation was done following the method that Diakou and Carde reported^27^ with modifications. The tissues were cut into small blocks (about 4–6 mm^3^), and immediately fixed in 100 mM phosphate-buffered saline (PBS, pH 7.2) with 2.5% (V/V) glutaraldehyde for 4 h at 4 °C with thorough degassing. After wash for three times with PBS, the tissue blocks were fixed with 1% (W/V) OsO_4_ in PBS for 2 h at 4 °C. Following another extensive rinse in PBS, the tissue was dehydrated in a graded ethanol series (30–100%) and 100% acetone. The tissue was infiltrated with the Spurr Embedding Kit for 24 h at 4 °C. Polymerization was conducted at 70 °C for 8 h. The specimen was first semi-thin sectioned to localize the epidermal cells under the light microscope before the proceeding of the ultrathin (~60–90 nm) section preparation. The ultrathin section was put on the copper-grids of 100 meshes covered with 0.5% formvar films which were stained first with 2% uranyl acetate in 60% ethanol for 15 min at 25 °C and then with alkaline lead citrate for 10 min. The ultrathin sections were investigated with a Hitachi H-7650 electron microscopy.

### Starch extraction

Starch was extracted according to the method in Xu *et al.*^28^ with slight modifications. The Samples (1 g fresh weight (FW) berry and 0.5 g FW leaf) were ground into powder in liquid nitrogen, and then were extracted in 5 ml or 2.5 ml 80% (V/V) ethanol at 70 °C for 30 min. The homogenate was centrifuged at 12,000 *g* for 15 min at 4 °C, and then the supernatant was removed and centrifuged at the same condition. The precipitate was re-extracted three times with 25 ml aliquots 80% (V/V) Ca(NO_3_)_2_ for 10 min at 100 °C, and the three extracts were made up to 25 ml in a volumetric flask. The 2 ml extract solution and 2 ml the standard starch solution was added respectively 0.01 mol L^−1^ I_2_-KI solution. The starch content was then measured at optical density 620 nm.

### Determination of carbohydrate content

Soluble carbohydrate of fruits and leaves were measured based on the method used by Niu *et al.*^29^ with slight modifications. Triplicate tissues were prepared for fruits and leaves at each stage of development. Frozen samples (about 3 g FW) were ground in liquid nitrogen and extracted in 70% (V/V) ethanol of 6 ml and was incubated in water at 35 °C for 20 min, then it was centrifuged (6,500 *g*, at 4 °C for 15 min), and the supernatant was transferred to a 25 ml flask, the volume was topped up with 80% ethanol. The ethanolic extract was concentrated by nitrogen gas and the concentrate was reconstituted with 1 ml of ultrapure water. The soluble carbohydrates were separated by HPLC using a CARBOSep CHO-620 CA carbohydrate column (6×250 mm, Shoko Co., Ltd., Tokyo, Japan) dissolved in double-distilled H_2_O, following the manufacturer's instructions, and a refractive index detector (Waters, Sunnyvale, CA, USA). The column temperature was 80 °C and the flow rate was 0.6 ml min^−1^ with water as the eluent. Sucrose, glucose, and fructose were determined through their retention times and quantified based on standards.

### Enzyme extraction and assay

#### Protein extraction

Flesh, skin (~1 g) and leaf samples (~0.5 g) were ground with a buffer at 1:8 (W/V) ratio of buffer. The extract buffer was composed of 50 mM Hepes-NaOH (PH 7.5), 10 mM MgCl_2_, 2.5 mM DTT, 1.0 mM EDTA, 0.05% (V/V) Triton X-100, 0.1% (W/V) BSA, 0.1% β-mercaptoethanol and 2% (W/V) in soluble PVPP. PVPP was needed in the extraction method of *V. vinifera.* L since extracts quickly browned in its absence.^30^ The homogenate was centrifuged at 12,000 g for 15 min, crude supernatant was dialyzed for 16 h with 25 mM Hepes-NaOH (pH 7.5) and 0.25 mM EDTA-Na_2_. The insoluble pellet was homogenized in two times in 10 ml extraction buffer and then suspended in 3 ml of 50 mM Hepes-NaOH (pH 7.5) and 0.5 mM EDTA-Na_2_. Cell wall composition used to assay the activity of insoluble invertase was washed in 200 ml scattered (1:40 v/v) extraction buffer minus PVPP. Extracts were transferred into pre-chilled vials and examined immediately. All the process mentioned above were implemented at 0–4 °C. All activities were proportional to the amount of extracts and to reaction time.

#### Assay of activity of α-amylase and β-amylase

Activity of amylase was assayed according to Hagenimana *et al.*^31^ using 0.25 ml of 100 mM phosphate buffer (pH 6.0), 0.25 ml enzyme extract and 0.5 ml of 1% (g mL^−1^) starch solution. The reaction mixtures were pre-incubated for 5 min at 40 °C and terminated by adding 1 ml NaOH (0.4 M). The produced reducing sugars concentration was determined using DNS reagent based on the method of Miller.^32^ Activity of α-amylase was determined by following the same procedure as for total amylase. The enzyme extract was first pre-incubated for 15 min at 70 °C to deactivate as to β-amylase. Activity of β-amylase was calculated by the difference between the total amylase activity and the α-amylase activity. The activity of amylase was defined as the amount of maltose produced per hour per kg fresh weight of berry and leaves tissues under the experiment conditions.

#### Acid invertase

The soluble acid invertase (AI) and neutral invertase (NI) activity were determined by the method of Lowell *et al.*^33^ with slight modification. The 0.3 ml enzyme extract was incubated for 40 min at 37 °C with pH 4.5 80 mM acetate-K_3_PO_4_, and 500 mM sucrose, or pH 7.5 80 mM acetate-K_3_PO_4_ and 500 mM sucrose in a total volume of 1 ml for acid or neutral invertase activity assays, respectively. Reaction was stopped at 30 min by adding 600 μL 1% (w/v) 3, 5-Dinitrosalicylic acid (DNS) at 100 °C for 5 min and then read absorbance displayed at 540 nm. The activity of invertase was defined as the amount of glucose produced per h per kg fresh weight of grape berry tissue in the experiment conditions.

#### Sucrose phosphate synthase and sucrose synthase

Activity of sucrose phosphate synthase (SPS) was determined by the methods of Zhang *et al.*^34^ involving 55 μl of reaction solution and 85 μl crude enzyme extract sample. The reaction solutions were composed of 0.5 M Hepes-NaOH (pH 7.5), 0.14 M MgCl_2_, 0.028 M EDTA-Na_2_, 0.112 M F-6-P (fructose-6-phosphate), and 0.042 M uridine diphosphate glucose. The mixture was incubated for 40 min at 37 °C, following then the reaction was terminated by adding 70 μl of 1.0 M NaOH. Non-reacted F-6-P was degraded by placing the tubes in 100 °C water for 10 min. After cooling, 0.25 ml of 0.1% resorcinol solution (W/V, dissolved in 95% ethanol) and 0.75 mL of 35% HCl (V/V) were added into the mixture and then the tubes were incubated at 80 °C water bath for 8 min. The procedure of the sucrose synthase (SUS) assay was completely same to that of SPS except the replacement of F-6-P with 0.084 M fructose in the reaction mixtures. The amount of sucrose produced from F-6-P was calculated finally under the utilization of the standard curve made using sucrose and absorbance value at A_520_. Enzymes activity was described as micromoles of sucrose or glucose generated per h per g fresh weight of samples.

### ADP-glucose pyrophosphorylase

For the assay of ADP-Glucose pyrophosphorylase based on the method of Tiessen *et al.*,^35^ with slight modification, the crude enzymatic extract and reaction buffer were conducted at 30 °C in totally volume of 450 μl including 50 mM Hepes-NaOH, pH 7.5, 6 mM MgCl_2_, 10 μLM Glc-1,6-bisP, 6 mM NADP^+^, 5 mM Na-PPi, 0.08 unit per mL phosphoglucomutase (from rabbit muscle), 0.07 unit per mL G-6-P dehydrogenase (from yeast), and 1.2 mM ADPG with 3 mM DTT. Parallel assays were conducted lacking either Na-PPi or ADPG. Blank activity was insignificant for at least 30 min and was reduced when detected.

### Cloning and analysis of genes related to the starch metabolism

Total RNA was isolated from different berry and leaf, following the modified SDS method.^36^ To isolate *APS*, *APL*, *GBSS* and *AMY* homolog genes from grape fruits, the corresponding full-length cDNA sequenced of *APS*, *APL*, *GBSS1* and *AMY* in *Arabidopsis thaliana* (TAIR: http://www.*Arabidopsis*.org/index.jsp, accession numbers AT5G19220, AT5G48300.1, AT1G32900.1, AT4G25000) as querying probes, a blast search performed on the National Center for Biotechnology Information (NCBI, http://www.ncbi.nlm.nih.gov/blast/) database and further search against the Grape genome browser database Genoscope website were done. The results showed that the predicted CDS of these genes in grape (XM_002281033.2, XM_002263219.2, XM_002278998.2, XM_002285177.1) were highly homologous to their genes in *Arabidopsis thaliana*. On Based on the predicted CDS sequences of these genes in grape, specific primers (Table 1) were designed using Primer Premier 5.0 and Oligo 7 software, and synthesized by Beijing Dingguochangsheng Biotechnology Co., Ltd. (Shanghai, China).

PCR products of genes were ligated into pMD19-T vector and then transformed into *E.coli* JM 109. The positive clones identified by PCR analysis were sequenced by Invitrogen Biotechnology Co., Ltd. (Shanghai, China).

The similarity between starch metabolism genes in grape and those in other plants was determined using NCBI BLAST. The obtained cDNA sequences were further blasted to verify the open reading frame (ORF) of the predicted cDNA sequences and BioXM (Version 2.6) was used to deduce the amino acid sequences of the four genes. Amino acid sequence of each gene was submited to ExPASy server (http://expasy.org/tools/protparam.html) to predict their characters such as molecular weight and theoretical pI (Isoelectric point). The catalytic domain of these four amino acid sequence were identified using the InterProScan program (http://www.ebi.ac.uk/interpro/). The phylogenetic tree was constructed through the neighbor-joining method using the MEGA version 6.0.

### Quantitative real-time PCR analysis

The gene-specific primers of *VvAPL*, *VvAPS*, *VvGBSS1* and *VvAMY* were designed based on the cloned sequences above (four ORFs), and that of *VvBMY*, *VvSuS1*, *VvSPS* were based on the partial cDNA using Primer Premier 5.0 and Oligo 7 Software, *VvNIN*, *VvcwIN*, *VvAIN1* and *VvAIN2* were in according to the study of Hayes *et al.*^37^ All amplified production of primer pairs (see Table 2) was a single with the expected size, and fragment sequence was checked by melt-curve analysis, agarose gel electrophoresis, and DNA sequencing. RT-PCR experiment was carried out using a SYBR green method on a Applied Biosystems 2720 thermal cycler. We tested the suitability of *actin* (LOC100232968), *ubiquitin* (LOC100253716), and *β-tubulin* (LOC100247828) as appropriate reference genes, and then *actin* was used for normalization in all experiments. Samples were collected in technical triplicates and the mean values of cycle threshold (Ct) were used to calculate the mean normalized expression level (and the Standard Error) of each gene in each cDNA tested relative to the reference gene by Q-Gene software.^38^

## Results

### Ultrastructural changes of skin and flesh cells

**Figure 1:**
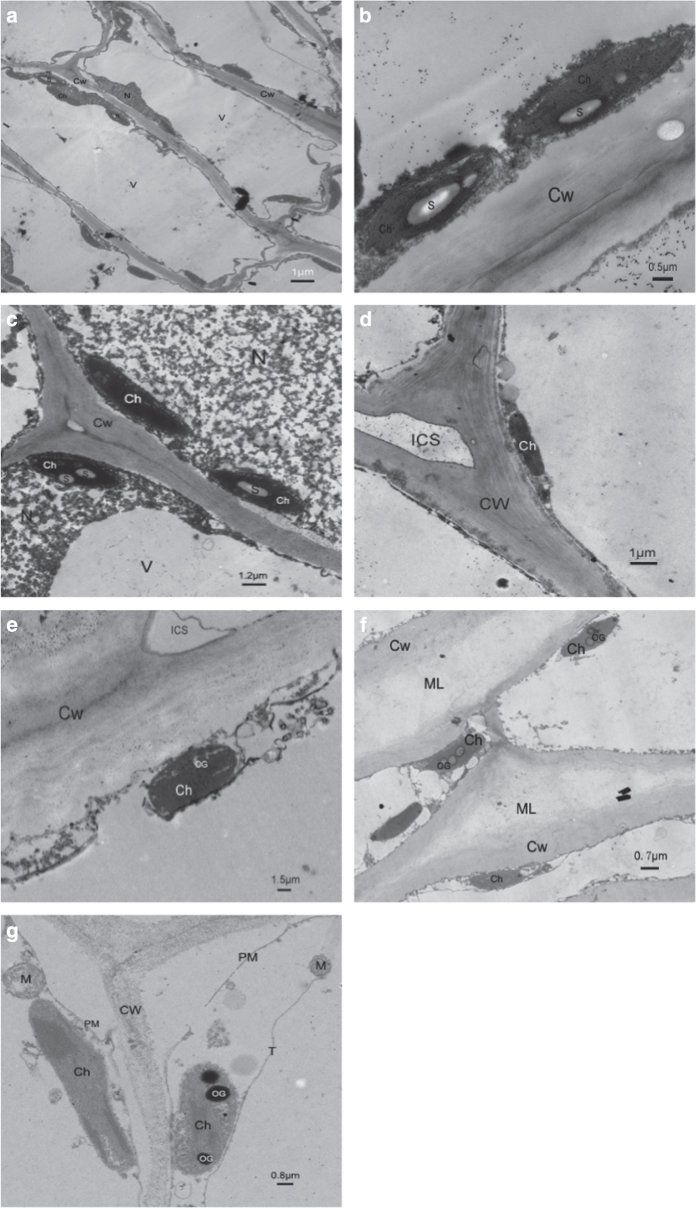
Ultrastructrural changes in the skin and flesh cells of grape berry at different development stage. (**a**) skin cell at the 14 DAA, bar=1 μm; (**b**) Chloroplast in skin cell at the 14 DAA, Bar=0.5 μm; (**c**) Flesh cell at the 14 DAA, bar=1.2 μm; (**d**) Skin cell at the 42 DAA, bar=1 μm; (**e**) Flesh cell at the 42 DAA, bar=1.5 μm; (**f**) Skin cell at the 70 DAA, bar=0.7 μm; (**g**) Flesh cell at the 70 DAA, bar=0.8 μm. CW, cell wall; Ch, chloroplast; ICS, intercellular space; M, mitochondrion; ML, middle lamella; N, nucleus; OG, osmiophilic globule; PM, plasma membrane; PP, polyphenolic; S, starch granule; T, tonoplast; V, vacuole; VE, vesicle.

The grape berries from three different developmental stages were sampled and observed via transmission electron microscopy (Figure 1). These three stages included stage I (the first rapid phase of berry growth, 28 DAA), stage II (the lag phase of berry growth, 42 DAA), stage III (the second rapid phase of berry growth and fruit ripening, 70 DAA), respectively. During the growth and ripening period of berry, the peripheral cells elongated tangentially, and radial width remained nearly constant. However, the flesh cells greatly enlarged to an irregular shape which is accompanied by the expansion of the central vacuole, which was of a same phenomenon as previous reports.^39,40^ At the same time, the tangential cell walls of the skins became thickened, and the flesh cell walls remained very thin, during which some profound changes in chemical composition occurred.^41,42^

Transmission electron microscopy (TEM) micrographs showed clear difference between the structures of the skin and the flesh cells. At stage I (28 DAA), the young berries were firm, both the flesh and skin cells were arranged closely, enriched in chloroplasts, and had a dense cytoplasm. In skin cells, most of the chloroplasts were distributed along the inner cell walls, presenting large starch granules that occupied 50% of chloroplast volume. The size of starch granules was about 1.5 μm at 14 DAA. At stage II (56 DAA), there appeared some osmiophilic granules in the flesh cells. The starch granules in the flesh cells disappeared soon and the cytoplasm became thinner. Meanwhile, the starch granules in the skin were hardly observed during the appearance of these osmiophilic granules possibly corresponding to tannins formed in tannosomes.^43^ Compared with the stage I and stage II, the electron density both of the berry flesh and skin cell walls during the ripening stage (stage III, 70 DAA) became lower and the space between cells changed to be larger, which might be associated with berry softening. In addition, the flesh cell cytoplasm was so thin that the chloroplasts were hard to observe at this stage.

### Cloning of genes related to starch metabolism

The fragments of each of the four genes (Table 1) cloned from fruit-derived cDNA were linked well together using BioXM software (Version 2.6), resulting in four sequences of 1,530, 1,563, 1,818, and 1,275 nucleotides encoding full-length ORF of, respectively, 509, 520, 605, and 424 amino acids for *VvAPL*, *VvAPS*, *VvGBSS1* and *VvAMY* genes in grape. Their GenBank accession numbers, predicted molecular weights and theoretical pIs are shown in Table 2. Clustal Omega (http://www.ebi.ac.uk/Tools/msa/clustalo/) was performed to study the amino acid sequences’ similarity between the genes of *APL*, *APS*, *GBSS1* and *AMY* in grape and those in other plant species. The analysis revealed that the similarities of amino acid sequences between the four genes in grape and those in other plant species were high (70–94%). It was found that *VvAPL*, *VvAPS*, *VvGBSS1*, and *VvAMY* shared the highest identities of 87, 94, 78, and 74% with ADP-glucose pyrophosphorylase large subunit of *Fragaria*×*ananassa* (Accession no. AY518345), ADP-glucose pyrophosphorylase of *Diospyros kaki* (Accession no. AB514525), granule-bound starch synthase of *Nelumbo nucifera* (Accession no. EU938541), secreted α-amylase in *Malus*×*domestica* (Accession no. AY939870), respectively. InterProScan analysis showed that the proteins deduced from *VvAPL* and *VvAPS* belonged to the same family, the Glucose-1-phosphate adenylyltransferase (InterPro acc. no. IPR011831) family also called ADP-glucose pyrophosphorylase, indicating that this enzyme was involved in the glycogen synthesis pathways in grape.^24^ *VvGBSS1* protein had a catalytic domain of starch synthase (InterPro acc. no. IPR013534) that used ADP-glucose (EC: 2.4.1.21), as in animals, as the glucose donor for starch synthesis. The analysis result revealed that *VvAMY* belonged to alpha-amylase family in plant (InterPro acc. no. IPR013775), and it hydrolyzed the glycosidic bond between two or more carbohydrates, or between carbohydrate and non-carbohydrate moiety (Table 3).

### Carbohydrate profiling of skin and flesh during grape berry development

**Figure 2:**
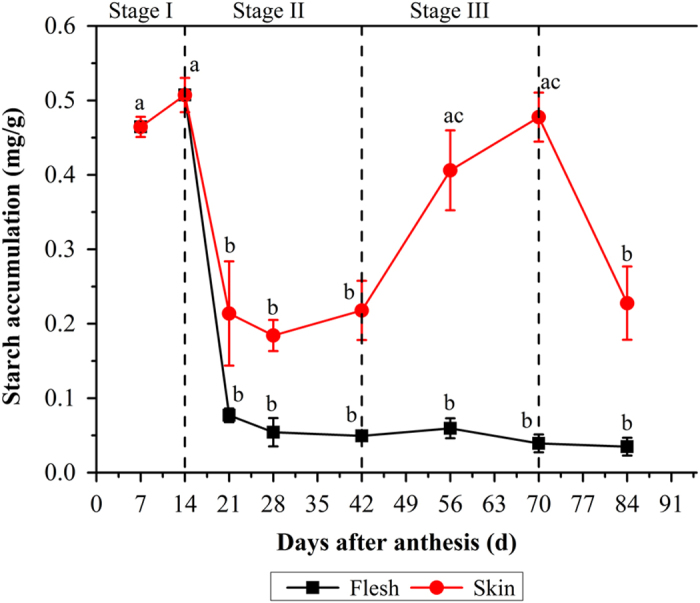
Changes of starch content in the flesh and skin during grape berry development. The vertical bar represents mean±s.e. (*n*=9). Bars with the same letters are not significantly different at *P*⩽0.05. Error bars smaller than symbol size are not visible.

**Figure 3:**
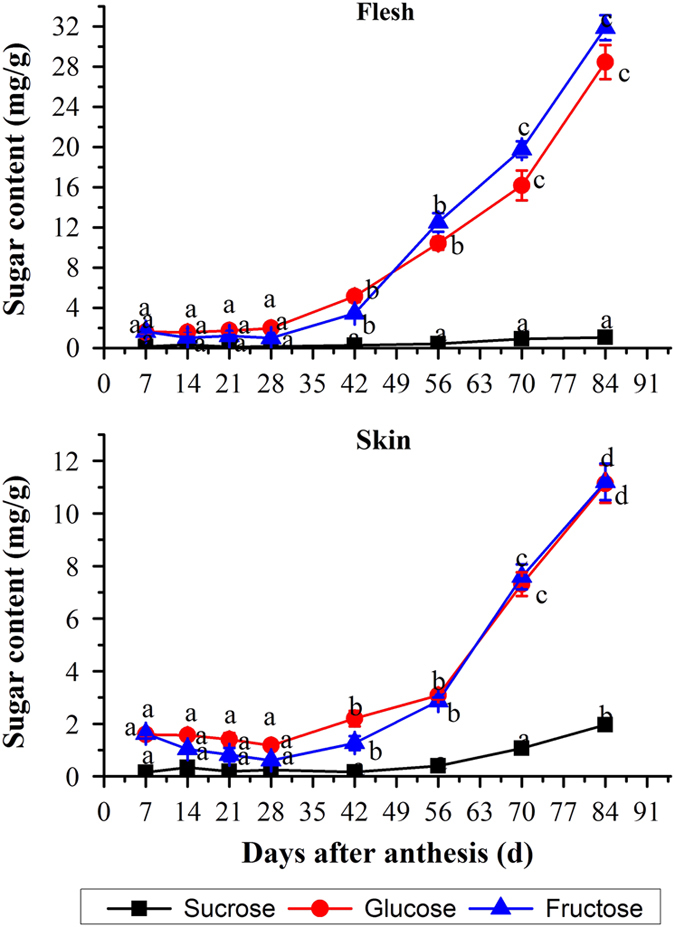
Changes in contents of the soluble sugar in skin and flesh throughout berry development. The vertical bar represents mean±s.e. (*n*=9). Bars with the same letters are not significantly different at *P*⩽0.05. Error bars smaller than symbol size are not visible.

There was an obviously distinct variation of the carbohydrate content in flesh and skin throughout the grape berry development (Figures 2 and 3). The berry developed from ovaries which were capable of storing starch at anthesis, and the starch was formed directly from photosynthesis in the chloroplasts as reported by Swift *et al.*^6^ At the early stages of growth and development, the starch content of berry kept a relatively high level and reached the highest concentration (0.507 mg/g FW) at around 14 DAA before gradually decreasing until the day of collection. However, the significant differences between the starch contents of flesh and skin were found from 21 DAA. The berry flesh established a remarkable decrease in the starch content and the concentration remained at a low level (<0.077 mg/g FW) until fruit ripening. Meanwhile, the skin always presented higher starch content than did flesh. From stage I to stage II, the starch content in the skin remained at a low level (0.184–0.217 mg/g), and then the starch content slightly increased to the second peak (0.317 mg/g FW) at véraison stage (56 DAA), followed by a significant decrease during fruit ripening. As shown in Figure 3, three kinds of sugar in grape berry not only exhibited same variation patterns, but also had comparable concentrations throughout berry development (except for the sucrose). Same as the previous reports, the glucose and fructose were the major soluble carbohydrates form in both flesh and skin of grape berry, and their contents both in flesh and skin showed a significant increase after 28 DAA, while the sucrose concentration remained at a low level at the early period of growth and development, and only increased slightly from 42 DAA until the day of collection.

### Analysis of enzyme activity in skin and flesh during grape berry development

**Figure 4:**
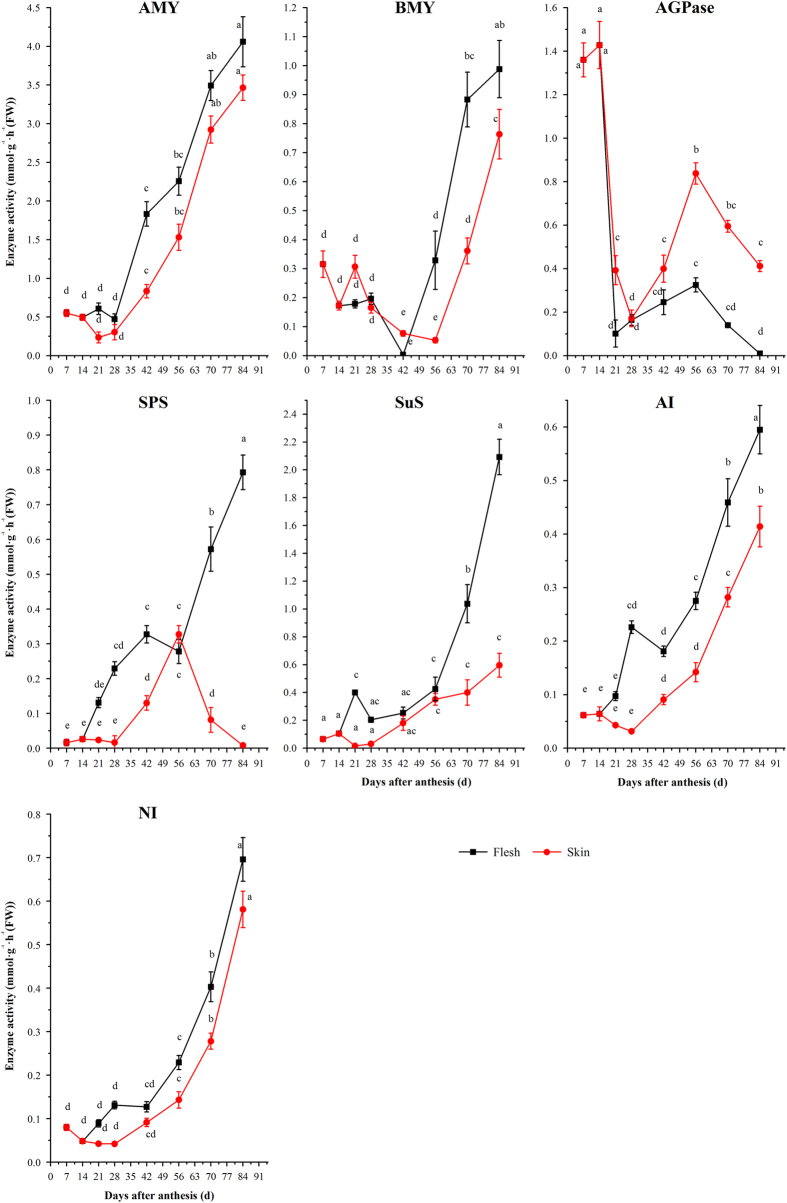
Changes of enzyme activities related to starch and sucrose metabolism in the flesh and skin during grape berry development. The vertical bar represents mean±s.e. (*n*=9). Bars with the same letters are not significantly different at *P*⩽0.05. Error bars smaller than symbol size are not visible.

Analysis of the activities of selected enzymes (AMY, BMY, SuS, AI and NI) in flesh and skin showed that they followed a similar pattern, where they all decreased slightly at the initial stage of berry growth and development, and then increased significantly around from 42 DAA until collecting time, with an exception concerning the activity variation profiles of AGPase and of the SPS in skin (Figure 4). It was obvious that the activities of most enzymes in flesh were higher than those in skin except for AGPase. The activity of AGPase in flesh and skin kept a high level during the initial stage of berry growth and development. After a decline from 14 DAA to 21 DAA, it increased significantly to the highest activity at 56 DAA, and then decreased significantly till to maturation of berry. In addition, the AGPase activity in flesh was lower than that in skin after 28 DAA. Interestingly, the SPS activity in flesh showed a similar change pattern as that of the activity of AGPase, while the SPS activity in skin increased with the fruit development before 42 DAA and then decreased significantly afterwards. Especially, the fact that the activity of AGPase in skin showed a high level after 56 DAA is consistent with the high content of starch in this tissue at the same stage.

### Expression patterns of genes related to starch and sucrose metabolism in the development of grape berry

**Figure 5:**
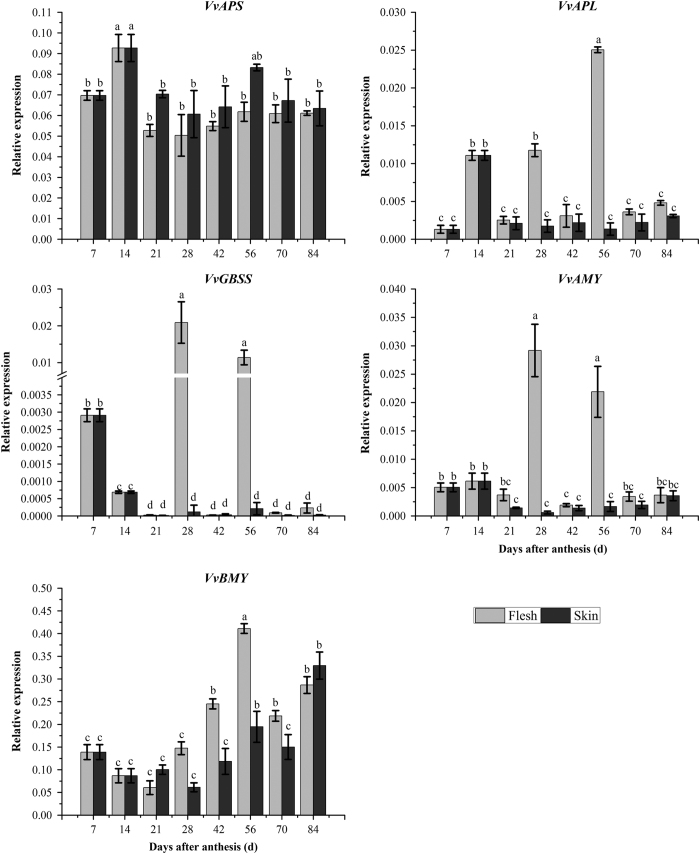
The gene expression related to starch metabolism in the skin and flesh during grape berry development. The vertical bar represents mean±s.e. (*n*=9). Bars with the same letters are not significantly different at *P*⩽0.05. Error bars smaller than symbol size are not visible.

**Figure 6:**
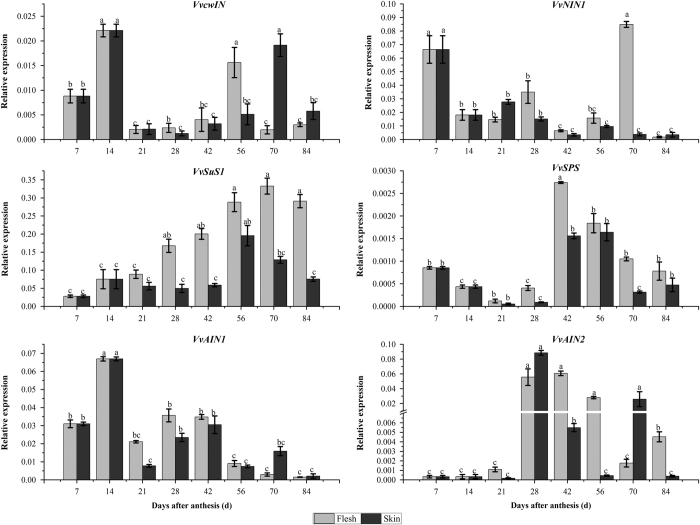
The gene expression related to sucrose metabolism in the skin and flesh during grape berry development. The vertical bar represents mean±s.e. (*n*=9). Bars with the same letters are not significantly different at *P*⩽0.05. Error bars smaller than symbol size are not visible.

In order to know the transcription profiles of genes related to starch and sucrose metabolisms in flesh and skin of grape berry and their roles in berry development, the transcript levels of the 11 genes were surveyed using the qRT-PCR technology. These 11 genes showed four different expression patterns (Figures 5 and 6). In flesh and skin, the expression of AGPase small subunit gene (*VvAPS*) had an obvious increase before 14 DAA and then decreased to a relatively lower level at 21 DAA that was kept constant until collection. However, the expression level of *VvAPS* in the skin keep steady during berry development, suggesting the more active role of *VvAPS* in starch accumulation there. In most of the time, the expression level of AGPase large subunit gene (*VvAPL*) was kept at low level in both the flesh and the skin compared to *VvAPL*. *VvAPL* was highly expressed in the flesh at 14 DAA, 28 DAA and reached the highest level at 56 DAA, but it was only highly expressed in the skin at 14 DAA. Another expression mode was those of granule-bound starch synthase 1 gene (*VvGBSS1*) and α-amylase gene (*VvAMY*). It was the situation that there were sharp increases to the detectable levels at 28 and 56 DAA in the flesh tissue, and then their expressions were almost undetectable in both the flesh and skin. The expression patterns of *VvBMY*, *VvSPS*, *VvSuS1* and *VvAIN2* were of the third situation. Their expressions seemed to coincide with the increasing contents of sucrose and hexose in both the flesh and the skin during the late development period. The expression of β-amylase gene (*VvBMY*) remained at a constant level up to 42 DAA followed by a significant increase, and then slightly decreased in both the flesh and the skin. The expression levels of *VvSPS* and *VvAIN2* in the two tissues showed similar pattern as that of *VvBMY* throughout the fruit development. The expression of *VvSuS1* both in the flesh and the skin increased until 56 DAA and its expression in the flesh kept at the constant high level with minor decrease before collection stage, while its expression level in the skin had a decrease after 56 DAA. The acid invertase genes (*VvAIN1, VvAIN2*) seemed to have similar functions, but their expression profiles were different from each other. *VvAIN1* was expressed at the highest level around 14 DAA, showed the profile as the second situation of *VvGBSS1* and *VvAMY*, while *VvAIN2* did the same as the gene group of *VvBMY* and *VvSuS11*. The cell wall invertase gene (*VvcwIN*) and neutral invertase gene (*VvNIN1*) were found highly expressed both in the flesh and the skin during the first phase and in flesh and/or the skin at the second rapid period of berry growth and development. Their expression patterns could suggest that they might play role for sugar accumulation at the early developmental stage.

### Relationships between gene expression, enzyme activities and carbohydrate content

In this study, the starch contents in the flesh exhibited a significant positive correlation with AGPase activity and with the relative expression levels of *VvAPS* and *VvAIN1*, but a negative correlation with the expression levels of *VvSuS1* (Table 4). Interestingly, the starch content in the skin also displayed a strong positive correlation to the activities of AGPase and SPS, and to the relative expression levels of *VvcwIN* instead of those of *VvAPS* and *VvAPL*. The contents of fructose, glucose, and sucrose both in the flesh and the skin all had positive correlation with activities of AMY, BMY, SuS and AI at a significantly level. In addition, their accumulation in the flesh had also significant positive correlation with the expression level of *VvSuS1* and the activity of SPS, and in the skin, with enzyme activity except for NI. *VvBMY* was the only gene which has significant positive correlation with these sugar concentrations.

### Circadian rhythm of carbohydrate accumulation

**Figure 7:**
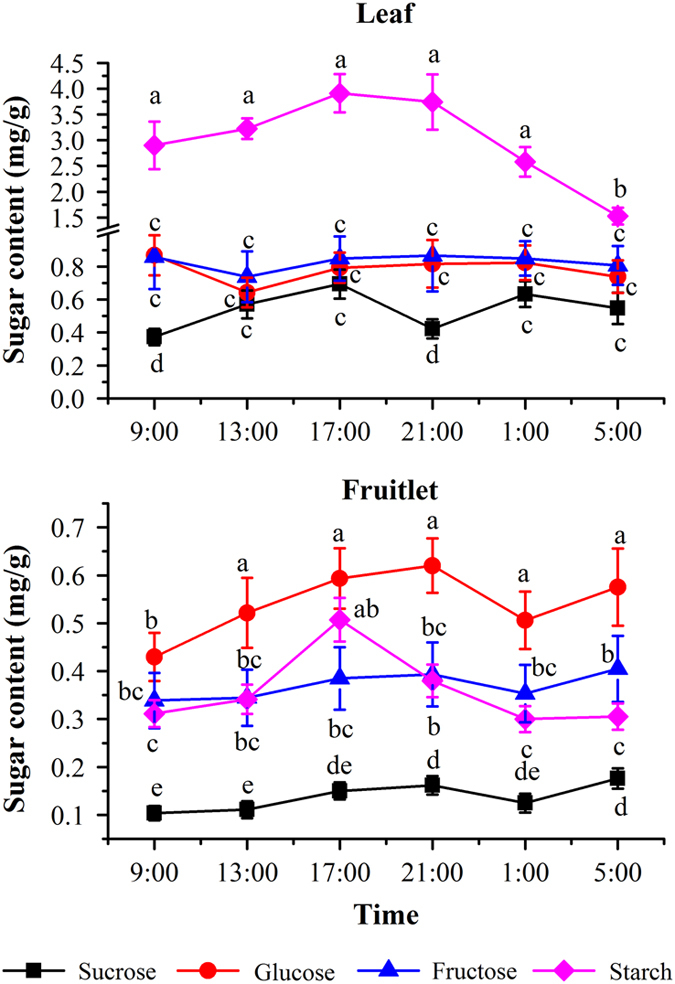
Circadian changes of the sugar contents in the leaf and fruitlet of grape. The vertical bar represents mean±s.e. (*n*=9). Bars with the same letters are not significantly different at *P*⩽0.05. Error bars smaller than symbol size are not visible.

For most of the plants, a large amount of the carbohydrates accumulated at the day is deposited transiently in the chloroplast in the form of starch and degraded at night into sugars to supply growth. To investigate the circadian rhythm of transitory starch in grape berry, the changes of starch levels in the leaves and fruitlets were measured both at day and at night times. We found that the starch concentration in the fruitlet was much lower than that in leaves (Figure 7). However, starch accumulation in the fruitlets and the leaves exhibited a similar circadian rhythm: the starch contents continuously increased to reach a highest level before 17:00 hours and then decreased at night to its lowest level at 5:00 hours next day. This phenomenon showed that starch was accumulated progressively at the daytime and gradually degradation at night. It was reported that much of the degraded sugars in leaves were transported into fruitlets as carbon sinks.^44^ Even though the content of starch in fruitlet was low, photosynthesis took place in the fruitlets, and there were some starch detected in fruit during the circadian cycle.^1^ Considering the water dilution effect,^45^ this results may be attributed to berry shrinkage during the daytime and increase in volume during the night, even a constant starch content can show higher concentration during the day and lower concentration during the night, as this paper observed. However, we cannot completely rule out the possibility of the circadian rhythm of sugar accumulation.

The distribution of carbon from the source leaf is controlled partly by carbohydrate metabolism in the source tissues (leaf), although carbohydrate metabolism in the sink tissues (roots, flowers, fruits) also affect transportation.^46^ It was found that this made the changes of sugar levels in the leaves and in the fruits showing different circadian rhythms (Figure 7). In the leaves, the changes of the contents of fructose and glucose were relatively mild except for sucrose. The contents in leaves showed a maximum level at 9:00 and a minimal one at 13:00, and then followed by slight decrease at night. However, the content of sucrose exhibited some changes in a circadian rhythm. During the daytime, the content of sucrose increased lightly from the lowest level (0.27 mg/g) at 9:00 to the highest level (0.69 mg/g) at 17:00. While at night the sucrose content in the leaves declined to the low level at 21:00, and afterwards slightly recovered, then decreased again. The contents of these three soluble sugars in the fruitlets accumulated in a high similar pattern that did not show some characteristic of a clear circadian cycle. It was noticeable that the contents of glucose, fructose and sucrose kept increasing at the daytime from 9:00 to 21:00 hours, and then fluctuated at night.

### Enzyme activity in the circadian rhythm

**Figure 8:**
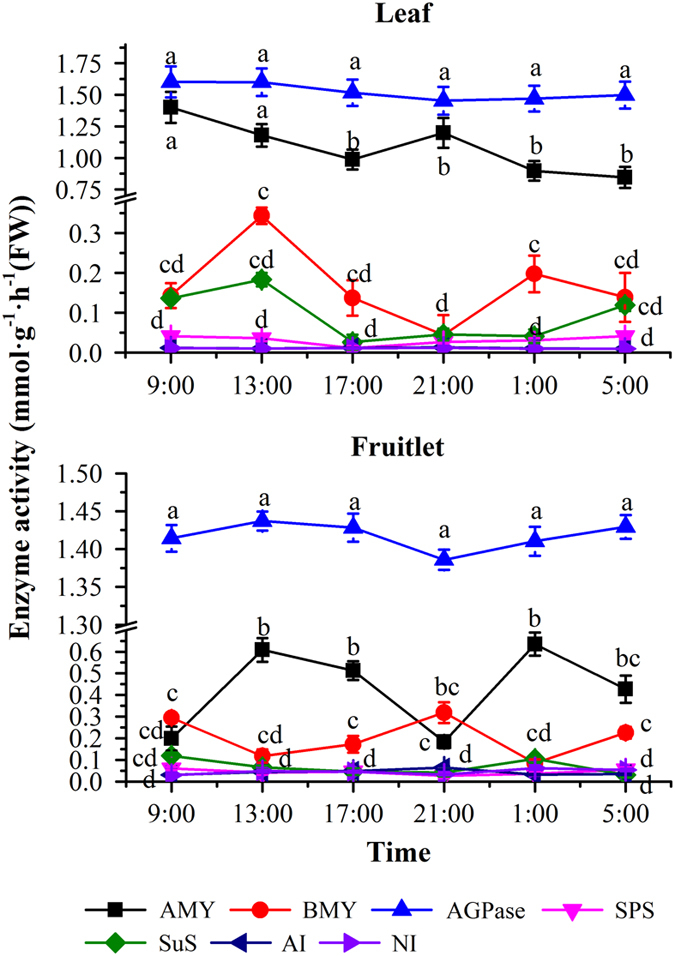
Circadian changes of enzyme activities related to starch and sucrose metabolism in the leaves and fruitlets. The vertical bar represents mean±s.e. (*n*=9). Bars with the same letters are not significantly different at *P*⩽0.05. Error bars smaller than symbol size are not visible.

To explore the effect of photoperiod on the activity of starch and sucrose metabolism enzymes, we measured the changes in enzyme activities during the day and night time. As shown in Figure 8, in both the leaves and fruitlets, AGPase, the first committed step in starch synthesis pathway, was very active at 13:00 hours and minimal at 21:00 hours. The activity of BMY in the leaves and fruitlets showed reverse varying patterns. At 13:00 hours, BMY activity increased to a highest level in the leaves, but it reduced to a lower level in the fruitlets. When BMY activity reduced to a lowest level in the leaves at 21:00 hours, it increased to a highest level in the fruitlets. The activity of AI exhibited a high level at 21:00 hours both in the leaves and the fruitlets. The activity of SPS reached to the lowest level at 17:00 hours in the leaves and at 21:00 hours in the fruitlets, respectively.

As a whole, the changes of AGPase and SPS activity displayed a clear circadian rhythm both in grape leaves and berry, which increased in daytime and declined at night. In addition, the changes of AI activity also displayed the circadian rhythm in the leaves and the berries, the highest level at night and the lowest level at daytime. In contrast, the starch degradation enzyme BMY not showed a clear circadian pattern.

### Gene expression variation during day and night time

To research the effect of photoperiod on the expression patterns of sugar metabolism related genes, we assayed the relative expression levels of these genes by quantitative real-time PCR (RT–PCR), of which the variation could imply the roles of the genes.

**Figure 9:**
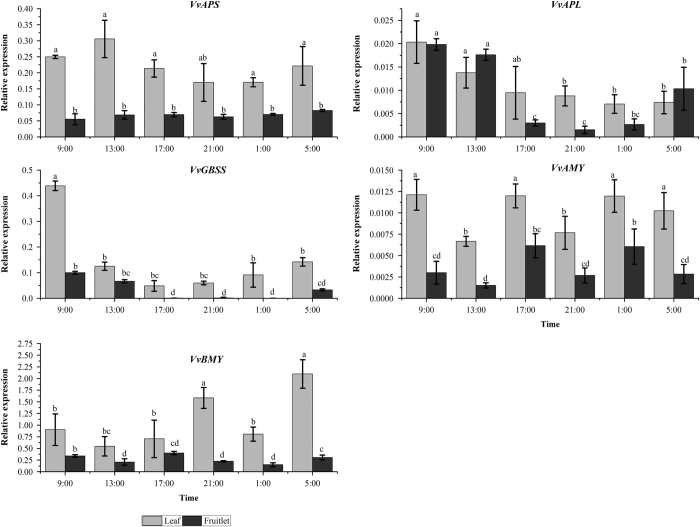
Circadian changes in the relative expression level of genes related to starch metabolism in the leaves and fruitlets. The vertical bar represents mean±s.e. (*n*=9). Bars with the same letters are not significantly different at *P*⩽0.05. Error bars smaller than symbol size are not visible.

Among the 11 genes we assayed, the 5 genes related to starch metabolism *VvAPS*, *VvAPL*, *VvAMY*, *VvBMY* and *VvGBSS1* obviously shared the same expression patterns between the leaves and fruitlets tissues, indicating the starch metabolism happened both in grape leaves and berry (Figure 9). However, the expression profiles of the 6 sucrose metabolism related genes revealed obvious difference between the leaves and fruitlets.

**Figure 10:**
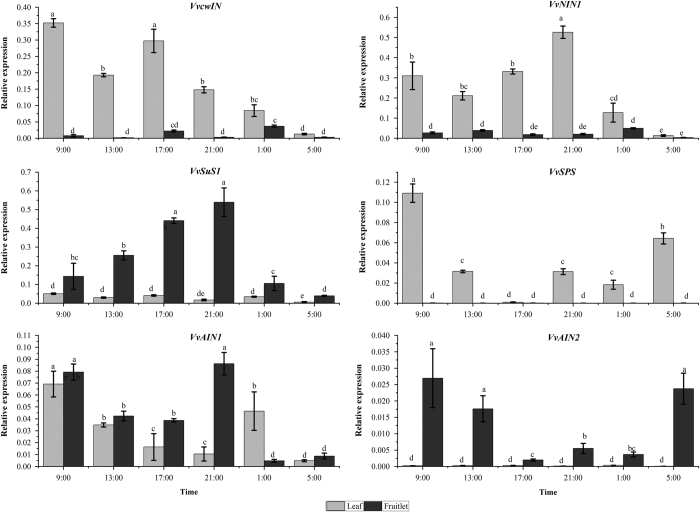
Circadian changes in the relative expression level of genes related to sucrose metabolism in the leaves and fruitlets. The vertical bar represents mean±s.e. (*n*=9). Bars with the same letters are not significantly different at *P*⩽0.05. Error bars smaller than symbol size are not visible.

In both the leaves and fruitlets, *VvGBSS1* and *VvAPL* expressed at high level in daytime and at low level in dark time. In addition, expression of *VvSuS1* increased at the daytime and then declined at night in the fruitlets, while *VvAIN2* also expressed at high level in daytime and at low level in dark time in the fruitlets (Figure 10). Similarity, the expressions of *VvSuS1* and *VvAIN2* were so low that they were not observed in the leaves compared to those in the fruitlets. Therefore, we concluded that these genes not displayed a circadian expression (Figure 10).

During the circadian rhythm, no correlation was observed between gene expression, enzyme activities, and carbohydrate concentration. Only in leaves, fructose accumulation had positive correlation with and BMY activity (Table 5).

## Discussion

Grape berries are known as a non-climacteric type of fleshy fruit without the drastically fluctuation of respiratory rate. Many fruits can accumulate abundant starch during fruit set and convert starch to sugars during ripening. In contrast, starch accumulated in fruitlets represents virtually nothing, when compared to sugar accumulated during ripening in grape berry. However, there are still some unknown on biological functions of starch in grape berry. In this study, the grape cultivar ‘Yong you 1 hao’ was used as the plant material by monitoring the developmental profiles of metabolites such as starch, glucose, fructose and sucrose to achieve a fully insights of the role of starch to the berry quality of grape and the relationship between transient starch and sugar accumulation during the circadian rhythm. Here it is confirmed that starch was predominantly localized in the chloroplast of skins and flesh cells at the early developmental stage of grape berry. We also observed that there was some trace of starch accumulated in skin cells at véraison. The genes of *VvAPS*, *VvSPS* and the enzymes of AGPase, SPS were the key ones that regulate the starch synthesis and the transformation of starch and sucrose throughout the development of grape berry, which exhibited a circadian rhythm mode. The increase of AMY activity maye indicate the start of fruit ripening and the high expression of *VvBMY* during the berry development, can suggest that AMY and BMY played a more important role in regulating starch degradation. The difference in the activities of SuS, AI, NI and the expression profiles of *VvSuS1*, *VvcwIN*, *VvAIN1*, *VvAIN2* and *VvNIN* between the skin and the flesh revealed that the flesh, as the sink organ, imported most of sugars compared to the skin, and the skin could also act as a metabolism center providing energy for synthesis of other metabolites and promote the accumulation of sugar in flesh at véraison.

### The role of starch in the development of grape berry

Starch is a good storage of carbohydrates widely synthesized in photosynthetic tissues of plant, such as leaf and skin, and in non-photosynthetic tissues, such as seed, root and tuber. Like melon and apple, starch accumulates during the initial stage of grape berry development, and then degraded mainly during ripening. With the berry development, starch is rapidly metabolized and is converted into soluble sugars, making it almost undetectable on grape berry at collection. Starch is one of the primary productions of photosynthesis occurred in the chloroplast and serves to buffer the changing availability of photosynthesis resulting from the light and dark cycle in a day. It plays a key role in the daily carbohydrate metabolism of the leaf. Accordingly, starch in leaf is regarded as a short-time carbohydrate pool and therefore is often called ‘transitory starch’.^7^ Among this study, a ultra-structure of the developing fruit revealed that the starch granules were localized in the green chloroplast of skin and flesh cells at the initial rapid growth phase of grape (stage I). Likewise, the starch grains gradually disappeared in the amyloplast with simultaneous appearance of osmiophilic globules filling up the amyloplast gradually.

**Figure 11:**
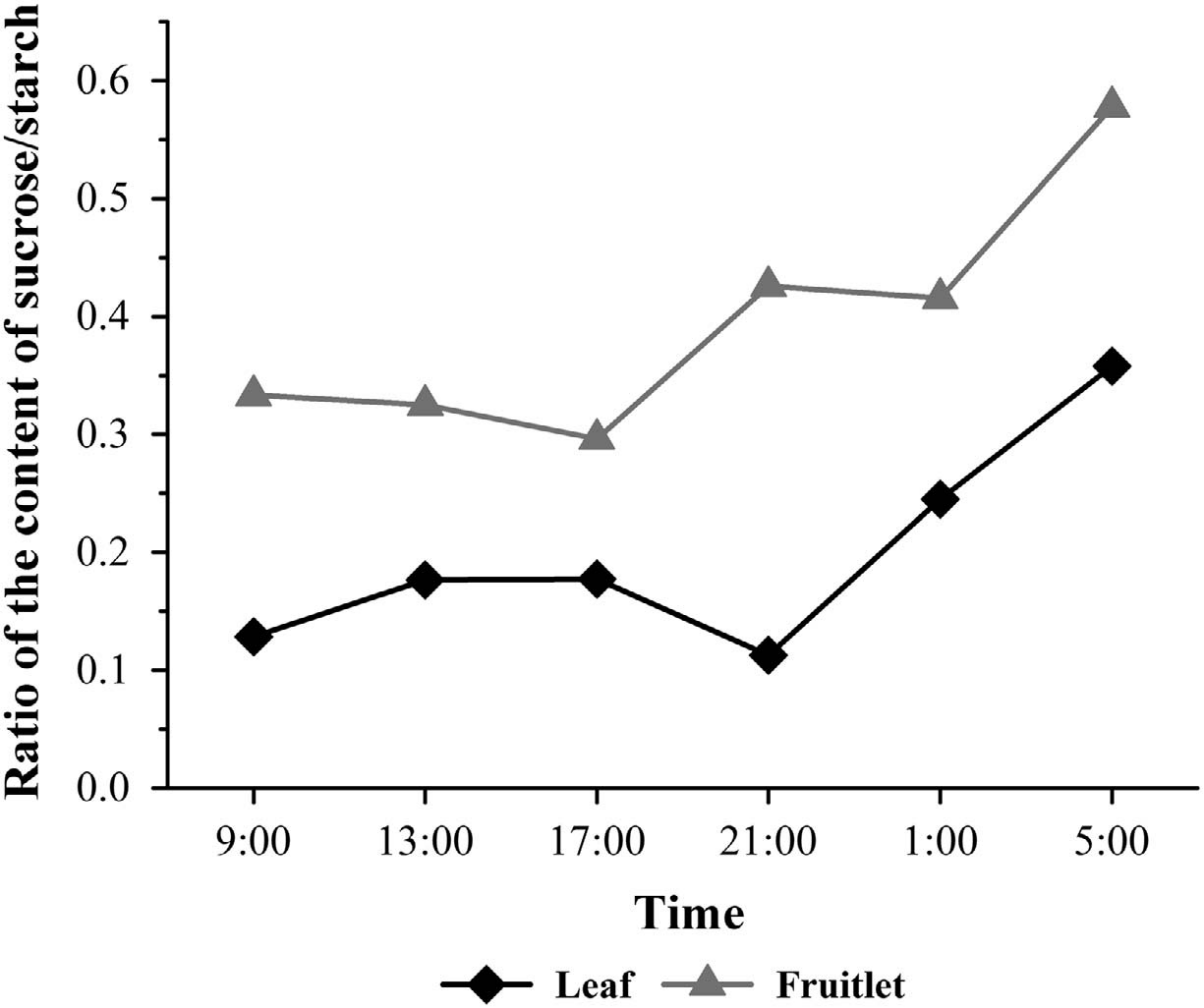
The ratio of content of sucrose/starch between the fruitlet and leaf during the circadian rhythm.

During the circadian rhythm, the ratio of sucrose/starch between the fruitlet and leaves exhibited similar time trends with little variation (Figure 11), suggesting that fruitlet itself possessed some weak photosynthetic capacity. The expressions of *VvAPL* and *VvGBSS1* in the leaf and fruit were regulated by light-dark cycles, indicating that these genes played the essential roles in starch biosynthesis during the grape berry development, and their expression levels could also suggest the ongoing of photosynthesis in grape fruitlets, more specifically, in the skin. Hunter *et al.*^1^ found that the starch content of leaves increased from the morning to the afternoon during the grape growth before veraison, indicating a percentage change in storage or transportation of carbohydrates between day and night. At later developmental stages the starch levels slightly declined or remained the same under the diurnal cycle. With fruit development and ripening, the ultrastructural changes in the skin and the flesh cells occurred and they were clearly observed with the transmission electron microscope. The microscope window showed that the chloroplasts kept degrading, the thylakoids disintegrating, and the starch granules disappearing gradually with the appearance of the cluster-like osmiophilic globules that was in larger size (Figure 1). The sudden generation of some trace amounts of starch detected in the skin at véraison stage indicated the involvement of starch as sugar source in the skin of grape berry. Véraison is the onset of grape berry ripening that indicates the initial of the second rapid growth stage and berry softening.^9^ Therefore, it can be concluded that even though there were only the trace amounts of starch in the grape berry, the starch present in the skin might participate to the grape berry coloring in the colored grape cultivars and ripening, not merely a source to supply energy and intermediate metabolite.

### Sugar metabolism in the skin and flesh during grape development

At the stage I (from 7 DAA to 28 DAA) of grape berry development, the berry cells underwent rapid division and expansion.^47^ The carbohydrate concentration in the flesh and the skin like hexose and sucrose remained at a relatively stable low level, while the starch content varied much as it decreased rapidly to a low level at 28 DAA. The starch content in grape berry flesh was much lower than that in the skin. During this period, the flesh was characterized by a dramatic reduction in the activities of AGPase, but the activities of AMY, BMY, SPS, SuS, AI and NI was observed to have a slight increase. In the skin, these enzymes exhibited lower activities, except for BMY and AMY. In fact, the expression profiles of genes related to sugar metabolism in the skin and in the flesh exhibited more differences. *VvSPS, VvSuS1, VvAIN1, VvAPL* and *VvAMY* in the flesh were expressed at higher level than they did in the skin. Among these 5 genes, *VvSuS1* exhibited the predominant expression that was consistent with the changes of the glucose and fructose contents in the flesh. This could be attributed to the function of SuS conducting the carbon allocation for cell wall biosynthesis, at the same time, this enzyme converted the imported sucrose into UDP glucose (UDPG) in sink organs, which is then changed into hexose-phosphate and ADP-glucose (ADPG) involved in the synthesis of starch.^48^ The high expression level of *VvSuS1* in our work may indicate a potential important role of VvSuS in cell wall synthesis and starch synthesis during the initial development phase of berry. Sarry *et al.*^49^ also proposed a futile sucrose recycling in the cytoplasm of grape berry, involving UDP glucose pyrophosphorylase and SuS. In the skin, the *VvAPS* and *VvAIN2* also showed high expression levels. The dramatic drop of starch in the flesh of grape berry was consistent with the previously described degradation in the starch during the early stage of strawberry fruit,^50,51^ together with the steady increase of the content glucose and fructose, the result may imply that the trait can be the signal for the beginning of sugar accumulation.

The stage II (from 28 DAA to 56 DAA) of grape berry development was the lag phase, during which berry size did not increase. It is well known that grape berry started to accumulate sugar around 45 DAA just after softening but possibly before color change.^52^ The variation in the activities of enzymes and expression patterns of genes involved in sugar metabolism were accompanied by the accumulation of sugar in both flesh and skin during this stage.^53^ The activity of AGPase, AMY, SPS, SuS and the expression of *VvAPS* and *VvSuS1* increased significantly in skin, together with the activity of AMY and the expression of *VvBMY* and *VvSuS1* in flesh. The starch content in the skin during the lag phase started to increase and finally reached a maximum at véraison, at least partially due to the role of AGPase and SuS. Moreover, both the expression and activities of acidic and neutral invertases (VvAI and VvNI) increased near the start of ripening and reached a peak in the later stage. This also was described by Zhang *et al.*^34^ After véraison, the development of grape berry enters the stage III that was represented by the initiation of coloring process, and accumulation continuously of sugar. During this period, the berry usually doubles its size. In addition to AGPase and SPS, all the enzyme activities increased rapidly both in the flesh and the skin. Previous studies indicated that the primary role of SuS was to degrade sucrose and supply simplicial glucose and fructose for many important metabolic processes.^54^ This could explain why in this study the expression of *VvSuS1* and SuS activity increased in the flesh with the fruit development, and meanwhile the amount of glucose and fructose in the berry continuously increase. This result was also consistent with some previous report on *Arabidopsis sus1/sus2/sus3/sus4* mutant,^48^ suggesting an important function of sucrose synthase in the normal cellulose and starch synthesis.^9^ In contrast, genes related to starch metabolism in the flesh, such as *VvAPL*, *VvGBSS1*, *VvAMY*, *VvBMY*, were expressed at high levels at véraison and finally caused the low content of starch in the flesh. This revealed that the low content of starch in the grape flesh was from the combined functions of the genes related to starch synthesis and degradation.

### Starch metabolism during day and nighttime

The carbon metabolism including starch metabolism during day and night time shows a circadian rhythm phenomenon. In many plants, starch accumulates in the daytime and degrades in the night almost linearly, with the starch accumulation can adaptively change which supply the sugar continuously even in long-dark circumstances.^55^ Changes in gene expression generally led to strong driving or damped alters of enzyme activities. In this study, starch content displayed a similar pattern both in grape leaves and fruitlets with a peak at 17:00 and the lowest content at 9:00. Here, it was shown that the high expressions of *VvAPL* and *VvGBSS1* both in leaves and fruitlets were more likely to contribute to starch accumulation in the early morning from 5:00 to 9:00 (Figure 7). The expression of *VvBMY* dramatically increased at 21:00, which might result in a reduction of starch content. In *Arabidopsis* rosettes, it was proposed that sugar depletion at the closure of the night activated a transient inhibition of carbohydrate utilization at the start of the ensuing light phase, resulting in short-term accumulation of sugar and expression of AGPase.^56^ Mutational analyses in *Arabidopsis* showed that most of the activity of AGPase in the leaf was the result of the *APS1* and *APL1* genes’ expression. The *APS1* mutations resulted in no determinate AGPase activity and starch content in the leaves of the plants (*adg1* mutants).^57,58^ The *APL1* mutations resulted in a significant reduction in the AGPase activity and starch content in the leaves of the plants (*adg2* mutants).^59,60^ Scheidig *et al.*^61^ studied the starch-overexpression phenotype in potato (*Solanum tuberosum*) leaves by the antisense method. The ability to degrade starch significantly decreased in potato leaves due to the lacking of the chloroplastic BMY, especially in the dark, suggesting that hydrolytic cleavage was the predominate pathway instead of the phosphory-lytic cleavage within the degradation process of temporary starch. In *Arabidopsis* leaves, there were nine *AtBMY* genes that showed different diurnal pattern at transcript levels.^21^ The expression pattern of *VvBMY* in this study was similar to that of the *AtBMY*. The AMY3 protein was the only known chloroplast-targeted AMY in *Arabidopsis* leaf, and the expression of *AtAMY3* gene increased in the daytime and decreased in the dark. However, it had been demonstrated that AMY3 is not essential for starch breakdown and other enzymes can compensate its absence.^62^ The diurnal patterns of *VvAMY* expression both in leaves and fruitlets were different from those of *AtAMY3*, but the AMY activity showed a similar pattern between grape and *Arabidopsis*, suggesting that the *VvAMY* cloned in this study might be not located in the chloroplast. In other plants, such as the sweet potato, the *GBSS1* gene was expressed well in the tuberous roots, leaves and stems, and the expression profile in leaves exhibited a circadian-regulated pattern which was dramatically expressed after 2 h treatment of light and almost completely disappeared at 12 h dark condition during the light/dark period.^63,64^ Compared to the expression pattern in the grape leaves, the expression of *VvGBSS1* in grape fruitlets was more similar with that of *GBSS1* in sweet potato leaves, which implied that *VvGBSS1* could affect the carbon allocation under the circadian regulation in grape berry.

## Conclusion

In this study, we found that ta relatively high starch contents could be detected in the skin at véraison stage. The enzymes (AGPase, SPS) and genes (*VvAPS* and *VvSPS*) regulated the starch synthesis in the skin, while the genes (*VvAMY* and *VvBMY*) and enzymes (AMY and BMY) regulated starch degradation in the flesh. During the circadian cycle, the expressions of genes (*VvAPS*, *VvBMY* and *VvSPS)* and the activities of enzymes (AGPase, BMY and SPS) were regulated by the circadian rhythms in the fruitlets.

## Figures and Tables

**Table 1:** Primer sequences and amplification characteristics designed for cloning of four genes in grape ‘Yong you 1 hao’

*Gene name*	*Sequence (5′–3')*	*Start and end of sequence*	*Size of amplified product (bp)*	*Tm [°C]*
*VvAPL*	ATGGCTGTGTCAACGGATGC	1–885	885	60
	CTTCTTTTCTGCCTCTTCCCTTG			
	ACAGCCGTGCTTCAGATTTCG	758–1563	827	58
	TCATATCACAAATCCATCCT			
*VvAPS*	ATGGCGAGCTTGTCTGCGCTCGGAGTAAC	1–1530	1530	63
	CTAGATGATGGTTCCACTAGGAAGCAAGG			
*VvGBSS1*	AAAATGGCGACTCTGACTGCTTCTC	1–1821	1821	63
	TCAAGGAGTGGCAACATTTTCCTTGGC			
*VvAMY*	AAGAATGGGTATTACTACTTCCCTC	1–1289	1289	61
	CAGACCCAGATTACTGTTTCTCCCAC			

**Table 2:** Primer sequences and amplification characteristics designed for qRT-PCR of 11 genes in grape ‘Yong you 1 hao’

*Primer*	*Primer 5′-3′*	*Amplicon length (bp)*	*Tm (°C)*	*NCBI accession*
*VvAPL*	AAACAAACCGCTCCTCAGATG	155	58	XM_002281033.2
	AGTCGACTTGCCTGAAACTAC			
*VvAPS*	TCGGGGACAATGTGAAGGT	109	58	XM_002263219.2
	GGCATCCTTGATTACCGTG			
*VvGBSS*	TGCTGCTTTGGGAGAGATG	223	58	XM_002278998.2
	TGGATGAGGTAGTCAATGGATAG			
*VvAMY*	ACCACAGGACTGCTGAGAAG	261	56	XM_002285177.1
	ATCGCCATCCAGCAAAGC			
*VvBMY*	AGATGAAGGAAGGGAAGGAT	152	58	XM_002285533.1
	AAGCAGGCACAGAAGAAAAC			
*VvNI*	GGCTTGGGAAGAGGACTATG	176	58	XM_002280426.2
	GTTGCCTAAACGACGGTAAAT			
*VvSuS1*	CTGGGGTTTATGGGTTCTG	149	58	XM_002275119.2
	AATGCCTCTGCCTTTTAGC			
*VvSPS*	ACGCTGGGCTGCTTCTAC	119	58	XM_002282772.1
	AGGGGATCAATTCTGGTTTC			
*VvcwIN*	ACGAATCATCTAGTGTGGAGCAC	236	58	(Hayes *et al.*^37^)
	CTTAAACGATATCTCCACATCTGC			
*VvAIN1*	CCATCTCCATCCCATCGTAACC	233	58	(Hayes *et al.*^37^)
	GGCTATCCAAGTTTCCAACCAACC			
*VvAIN2*	GAGCACAGTTCCAGTAATCAAAGG	266	58	(Hayes *et al.*^37^)
	GTGAGGCGTAGTTTTAGGACTCC			
*UBI*	AGTAGATGACTGGATTGGAGGT	150	58	
	GAGTATCAAAACAAAAGCATCG			

**Table 3:** Basic information of genes cloned from the fruits of grape ‘Yong you 1 hao’

*Gene name*	*Size of amplified product (bp)*	*GenBank accession no.*	*No. of amino acid*	*Molecular weight (Da)*	*Theoretical pI*
*VvAPL*	1,563	KJ023683	520	57971.3	9.04
*VvAPS*	1,530	KF986867	509	55900.7	6.53
*VvGBSS1*	1,818	KF986868	605	65960.7	7.52
*VvAMY*	1,275	KF990163	424	47155.1	5.55

**Table 4:** Correlation coefficients between enzyme activities (mmol h^−1^ g^−1^ FW), expression level of genes related to starch and sucrose metabolizing enzymes and carbohydrate content (mg g^−1^) in the flesh and skin during grape berry development

	*Enzyme activity*							*Relative expression level*										
	*Starch metabolism*			*Sucrose metabolism*				*Starch metabolism*					*Sucrose metabolism*					
	*AGPase*	*AMY*	*BMY*	*SuS*	*SPS*	*AI*	*NI*	*VvAPS*	*VvAPL*	*VvGBSS1*	*VvAMY*	*VvBMY*	*VvSuS1*	*VvSPS*	*VvAIN1*	*VvAIN2*	*VvcwIN*	*VvNIN*
*Flesh*																		
Starch	**0.738**^a^	−0.555	−0.29	−0.476	−0.407	−0.623	−0.433	**0.846**^b^	−0.103	−0.213	−0.212	−0.492	−**0.745**^a^	−0.295	**0.791**^a^	−0.443	0.682	0.204
Glucose	−0.667	**0.993**^b^	**0.885**^b^	**0.921**^b^	**0.954**^b^	**0.965**^b^	0.406	−0.182	−0.017	−0.266	−0.229	0.636	**0.865**^b^	0.223	−0.558	−0.176	−0.312	0.009
Fructose	−0.429	**0.983**^b^	**0.922**^b^	**0.928**^b^	**0.958**^b^	**0.958**^b^	0.404	−0.142	−0.038	−0.285	−0.248	0.600	**0.832**^a^	0.157	−0.553	−0.258	−0.297	0.063
Sucrose	−0.214	**0.943**^b^	**0.925**^b^	**0.914**^b^	**0.961**^b^	**0.927**^b^	0.359	0.032	−0.069	−0.341	−0.299	0.480	**0.773**^a^	0.066	−0.370	−0.326	−0.193	0.073
																		
*Skin*																		
Starch	**0.854**^a^	0.094	−0.169	0.062	**0.721**^a^	0.012	−0.106	0.670	0.420	0.493	0.648	−0.064	0.344	0.134	0.479	−0.350	**0.839**^b^	0.299
Glucose	−0.258	**0.973**^b^	**0.773**^a^	**0.932**^b^	−0.365	**0.996**^b^	**0.985**^b^	−0.299	−0.103	−0.308	0.044	**0.868**^b^	0.260	−0.106	−0.494	−0.173	0.156	−0.465
Fructose	−0.215	**0.977**^b^	**0.775**^a^	**0.928**^b^	−0.333	**0.994**^b^	**0.979**^b^	−0.284	−0.117	−0.256	0.069	**0.865**^b^	0.274	−0.117	−0.496	−0.173	0.187	−0.416
Sucrose	−0.244	**0.930**^b^	**0.816**^a^	**0.893**^b^	−0.408	**0.972**^b^	**0.982**^b^	−0.262	−0.007	−0.314	0.104	**0.854**^b^	0.201	−0.207	−0.440	−0.124	0.162	−0.453

a Significant at *P*<0.05.

b Significant at *P*<0.01.

The number in boldface indicates that the value is statistically significant.

**Table 5:** Correlation coefficients between enzyme activities (mmol·h^−1^·g^−1^ FW), expression level of genes related to starch and sucrose metabolizing enzymes and carbohydrate content (mg/g) in the leaves and fruitlets during the circadian rhythm

	*Enzyme activity*				*Relative expression level*					
	*Starch metabolism*		*Sucrose metabolism*		*Starch metabolism*				*Sucrose metabolism*	
	*AGPase*	*BMY*	*SPS*	*AI*	*VvAPS*	*VvAPL*	*VvGBSS1*	*VvBMY*	*VvSPS*	*VvAIN1*
*Leaf*										
Starch	0.042	−0.109	−0.735	0.784	−0.036	0.164	−0.249	−0.758	−0.459	0.004
Glucose	−0.296	−0.725	−0.146	0.588	−0.648	0.160	0.380	−0.682	0.291	0.374
Fructose	−0.512	−**0.857**^a^	−0.347	0.649	−0.796	−0.071	0.122	−0.726	0.080	0.099
Sucrose	−0.221	0.358	−0.567	−0.450	−0.064	−0.589	−0.668	0.087	−0.787	−0.322
										
*Fruitlet*										
Starch	0.071	0.019	−0.197	0.582	−0.047	−0.193	−0.441	−0.464	0.077	0.196
Glucose	−0.204	0.117	−0.548	0.763	0.477	0.108	−0.770	−0.703	−0.589	−0.057
Fructose	−0.171	0.325	−0.161	0.446	0.602	0.330	−0.621	−0.803	−0.320	−0.154
Sucrose	−0.183	0.288	−0.179	0.406	0.640	0.391	−0.642	−0.765	−0.365	−0.211

a Significant at *P*<0.05.

The number in boldface indicates that the value is statistically significant.
